# Red musical identity and subjective wellbeing: a longitudinal study of the chain mediating roles of awe and prosocial behavior

**DOI:** 10.3389/fpsyg.2025.1635179

**Published:** 2025-10-06

**Authors:** Yongcan He, Shuo Wang, Binyue Liu, Pingping Wang, Jimei Yang, Maoping Zheng

**Affiliations:** ^1^Key Laboratory of Cognition and Personality (Ministry of Education), Southwest University, Chongqing, China; ^2^School of Psychology, Southwest University, Chongqing, China; ^3^School of Music and Dance, Zunyi Normal University, Zunyi, China; ^4^School of Music, The Chinese University of Hong Kong, Shenzhen, China; ^5^School of Educational Sciences, Anshun University, Anshun, China; ^6^School of Music, Southwest University, Chongqing, China

**Keywords:** red musical identity, subjective wellbeing, awe, prosocial behavior, longitudinal study

## Abstract

**Objectives:**

Red music, as a distinctive form of red culture in China, plays an important role in fostering national identity and promoting subjective wellbeing. However, empirical research regarding red musical identity remains scarce. This study addresses this gap through a longitudinal study, using three waves of data to explore the relationship and potential psychological mechanisms between red musical identity and subjective wellbeing.

**Methods:**

The Red Musical Identity Scale, Dispositional Positive Emotion Scale (Awe subscale), Prosocial Tendencies Measure, and Subjective Happiness Scale were used for assessments. The participants were 586 college students from a university in western China, the majority of whom were female (77.2%).

**Results:**

The results showed that red musical identity at Time 1, awe at Time 2, prosocial behavior at Time 2, and subjective wellbeing at Time 3 were significantly correlated, and all were positively associated with subjective wellbeing at Time 3. However, the direct effect of red musical identity at Time 1 on prosocial behavior at Time 2 was not significant. After adjusting for age, sex, awe, and prosocial behavior at baseline, red musical identity at Time 1 positively predicted subjective wellbeing at Time 3. Furthermore, awe at Time 2 mediated the path from red musical identity at Time 1 to subjective wellbeing at Time 3. In addition, a sequential indirect pathway was supported: red musical identity at Time 1 showed indirect effects on subjective wellbeing at Time 3 successively via the awe at Time 2 and prosocial behavior at Time 2.

**Conclusion:**

These findings provide preliminary longitudinal evidence for the psychological mechanisms linking red musical identity and subjective wellbeing. However, given the single-site, predominantly female student sample, the conclusions should be viewed as tentative and are most applicable to similar university contexts.

## 1 Introduction

Subjective wellbeing is an important metric for evaluating the quality of life of individuals and societies (Diener, 2000). In recent years, a number of studies have highlighted the potential importance of school education, particularly aesthetics education, in supporting wellbeing (Ye et al., 2025). The overarching objective of aesthetics education is to expand individuals’ cultural literacy by cultivating an understanding of aesthetic creation, with the ultimate aim of evoking profound emotional responses. Red music contributes to this education, embodying a noble revolutionary spirit and strong cultural heritage. It functions as a key conduit for promoting national spirit and cultural confidence. Research indicates that in higher education institutions, red music, as an aesthetic education tool, has been associated with students’ personal growth and with a stronger sense of identity (Han and Guo, 2023). Investigating the relationship between red musical identity and subjective wellbeing may contribute to a better understanding of societal mental health and wellbeing, and may offer useful insights for cultural and educational strategies. Therefore, this study employed a three-wave longitudinal design to examine the relationship between university students’ red musical identity and subjective wellbeing, aiming to provide preliminary evidence that could inform aesthetic education practices and contribute to discussions on musical literacy and subjective wellbeing.

### 1.1 Red musical identity

Red music is a distinctive form of red cultural heritage, capturing both the spirit of the times and national characteristics developed by the Communist Party of China as it united and led Chinese people of all ethnic groups in pursuit of national independence, liberation, prosperity, and wellbeing (Qi, 2023). Over time, red music has respond to meet the social and historical needs of different eras. From The March of the Volunteers (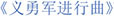) during the revolutionary wars to contemporary works like China in the Lantern Light (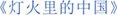), these songs have captured the spirit of their times, conveying patriotic sentiment and revolutionary ethos through stirring melodies and evocative lyrics, thereby reflecting and promoting the spirit and character of the Chinese nation (Zhang, 2024). Because music functions both as cultural expression and as a medium for identity formation (Hargreaves et al., 2002; Bowman, 2004), red music’s social significance is best examined through the concept of musical identity. Musical identity refers to a dynamic, socially constructed aspect of the self that develops through individuals’ emotional, cognitive, behavioral, cultural, and social engagement with music, shaping how they perceive and present themselves in cultural and social contexts (Hargreaves et al., 2002; Spychiger and Hechler, 2014; Bowman, 2004; Evans and McPherson, 2014). Building on this framework, red musical identity can be conceptualized as a culturally specific form of musical identity in the Chinese socio-historical context, characterized by individuals’ engagement with red music across three interrelated dimensions. This study defines red musical identity as an individual’s deep recognition and internalization of red music and the cultural values it represents, expressed across the cognitive-emotional, cultural and behavioral dimensions. The cognitive-emotional identity refers to an individual’s understanding and appreciation of the role red music plays in shaping socialist culture and promoting spiritual development, as well as their emotional resonance and sense of responsibility toward the historical significance of red music. Through this dimension, individuals experience the sacrifices and heroism of revolutionary martyrs, fostering patriotic emotions and positive psychological empowerment. Cultural identity refers to the enhancement of red music to reflect China’s revolutionary history and spirit, thereby deepening individuals’ understanding of red history, fostering patriotic emotions, and cultivating a positive psychological state of empowerment. Behavioral identity refers to an individual’s active engagement in learning, performing, and sharing red music, through which they consciously disseminate their historical and cultural values to a broader audience, thereby supporting and promoting red music culture through concrete actions. Historically, red music has contributed to processes of national consensus and collective memory, which have supported the emergence of what scholars term “red musical identity” (Guo, 2023). Taken together, red musical identity can be understood as a culturally specific sub-component of national identity, rooted in the Chinese historical context, while simultaneously functioning as a distinct form of musical identity in terms of its cultural expression and psychosocial mechanisms.

Red musical identity plays a critical role in fostering interaction between individual musical consciousness and collective social values. Research has shown that university students possess a sincere emotional connection to red culture and demonstrate relatively strong identification with its values. The integration of red music education into the college curriculum has been shown to cultivate students’ collective consciousness and patriotic feelings (Luo et al., 2016). By strengthening cultural identity and a sense of national pride, red music not only promotes interaction between individuals and society, but also enhances social cohesion and overall national wellbeing. The music promotes social interaction and emotional support through group activities (e.g., red music festivals), and may improve mental health and wellbeing through educational activities that convey historical knowledge and values. These help to create positive worldviews and strategies for coping with life challenges. Nevertheless, as society continues to evolve, public understanding of red music and its associated identity remains limited. On the one hand, as the public listens to, appreciates, and aesthetically neglects red music, it has led to a weakening of red musical identity, the sense of identity of red music decreases, and it is increasingly difficult to fully appreciate the profound historical and cultural connotations it contains. On the other hand, with the passage of time and social changes, it causes insufficient connection between red music and contemporary social issues and modern people’s life practices, ignoring the relevance of red music to the development of modern society, and thus neglecting the value of red musical identity in modern society. In light of these concerns, the present study investigated the relationship between university students’ red musical identity and subjective wellbeing, aiming to offer both theoretical insights and practical implications that may help inform approaches to support their musical literacy and subjective wellbeing.

### 1.2 The relationship between red musical identity and subjective wellbeing

Subjective wellbeing is defined as the overall emotional and cognitive evaluation of an individual’s quality of life, and is considered a significant indicator of quality of life for both individuals and society (Diener, 2000). Numerous studies have demonstrated the significant impact of cultural and artistic activities on subjective wellbeing (Blessi et al., 2016; Daykin et al., 2018; Mundet et al., 2017). Music has attracted considerable attention as a means of regulating emotions, eliciting positive and pleasurable experiences, promoting physical and mental health, and enhancing wellbeing (Rehfeldt et al., 2021). Various studies have demonstrated its association with subjective wellbeing. For instance, an intervention study that incorporated Chinese national music lessons found an increase in subjective wellbeing in individuals with low-to-moderate levels of wellbeing (Fu and Tu, 2023). The provision of music courses in higher education institutions aids aesthetic musical appreciation and identification among students (Behr et al., 2016). Although previous studies have not explored the relationship between red musical identity and subjective wellbeing, they have discussed the relationship between music identity and subjective wellbeing. Research has demonstrated that music can stimulate positive physical, psychological, and social outcomes in immigrant groups by engendering a sense of collective identity, promoting cultural preservation, and elevating social support and wellbeing. These serve as a conduit for the exchange of emotions and ideas (Rehfeldt et al., 2021). A recent study found a significant positive correlation between Chinese ethnic identity and wellbeing and that social support mediated the relationship between Chinese ethnic identity and wellbeing, which can effectively serve as a psychological buffer against the adverse effects of risk-related events, thereby facilitating the reconstruction of an individual’s sense of wellbeing (Shen et al., 2023). Moreover, some scholars argue that cultural identity should encompass both ethnic and national identity and that it has a broader scope, greater inclusiveness, and higher generality than ethnic identity (Phinney et al., 2001). Similar to ethnic identity, musical identity is regarded as having a significant influence on subjective wellbeing. However, it should be noted that musical identity is not restricted to any particular ethnic or cultural group; rather, it encompasses an individual’s identity with specific musical styles, genres, or cultural contexts. While studies such as Rehfeldt et al. (2021) have established a general positive association between musical identity and subjective wellbeing, these studies mainly examine broad musical categories or specific demographic groups and do not address musical forms that are deeply embedded in a nation’s political and historical context. The present study therefore focuses specifically on red musical identity within the Chinese collectivist cultural framework, emphasizing its historical roots, symbolic meanings, and potential to evoke distinct psychological states such as awe and prosocial motivation. This culturally grounded focus extends musical identity research into a historically important and value-laden musical form, and enhances practical relevance for culturally informed subjective wellbeing promotion. As a historical embodiment of the Communist Party of China’s revolutionary spirit, red music naturally invokes a sense of historical and social responsibility when individuals perform emotionally meaningful works through various modes of musical expression (Zheng, 2021). Listeners often deepen their sense of self-identity unconsciously when experiencing symbolically rich red music in any setting, thereby improving their subjective wellbeing. Red music lyrics and melodies are typically imbued with motivational and inspiring power, offering emotional comfort and helping individuals maintain an optimistic outlook when facing life pressures and challenges. This form of psychological identity plays a vital role in sustaining social wellbeing. Therefore, red musical identity may influence how individuals identify with Chinese culture, which in turn can impact their overall wellbeing. However, research remains limited on the specific effects of red musical identity on individual wellbeing and the potential mediating mechanisms involved. Given that red music can evoke strong emotions such as awe and foster prosocial tendencies, the connection between red musical identity and subjective wellbeing is worth examining. Awe is typically elicited by a sense of vastness which challenges existing perceptions. This sense may be sparked by objects of a grand and epic nature, such as symphonies, cathedrals, or music with unexpected harmonies and sudden dynamic shifts (Keltner and Haidt, 2003; Grewe et al., 2011). Recent experimental research with children further demonstrates that awe-eliciting art can increase individuals’ willingness to forgo self-interest in order to help others, including out-group members, and that such effects are accompanied by physiological changes linked to social engagement (Stamkou et al., 2023). Such awe-eliciting experiences may also augment prosocial motivation, encouraging behavior that benefits others, namely cooperation, helping, and altruistic actions (Piff et al., 2015; Stellar et al., 2017). By emphasizing collective values and shared emotional resonance, red music may similarly promote prosocial behavior, which has been consistently linked to enhanced wellbeing (Layous et al., 2013; Weinstein and Ryan, 2010). Based on this rationale, this study proposes Hypothesis 1: Red musical identity at Time 1 positively and significantly predicts subjective wellbeing at Time 3.

### 1.3 The mediating role of awe

Awe is a complex emotion involving wonder in the face of stimuli or objects beyond the scope of one’s cognition (Dong et al., 2013). Studies have shown that awe has positive mental health benefits. Besides encouraging cooperation and helpfulness (Piff et al., 2015), awe can allow people to view their lives more positively, and help them form closer interpersonal relationships (Rudd et al., 2012; Van Cappellen and Saroglou, 2012). As individuals frequently experience awe while immersed in musical or natural settings, participation in arts-related education has been shown to support this emotional response (Dong, 2016). Meta-analyses have demonstrated that music has positive effects on adults’ physical and psychological stress, pain, mental disorders, and overall wellbeing (Pelletier, 2004; Daykin et al., 2018; Laukka, 2007). While music can evoke positive emotions and improve wellbeing across diverse contexts, its emotional impact is also shaped by the cultural values and social structures in which it is embedded.

In the Chinese collectivist context, cultural expressions that highlight shared history, collective struggle, and group achievement are often effective in eliciting other-oriented awe, as such themes align with the kinds of social and collective stimuli shown to evoke awe in collectivist cultures (Bai et al., 2017; Keltner et al., 2022). Within such cultural contexts, music occupies a unique position in fostering collective sentiment. As such, red music draws upon revolutionary narratives and national history, integrating lyrical content that praises heroic figures and revolutionary ideals with melodic and structural features particularly suited to eliciting awe. Awe-inspiring musical features typically include unexpected harmonic shifts, abrupt dynamic changes, shifts in spatial or auditory perspective, and dramatic crescendos that generate intense aesthetic sensations and challenge listeners’ thinking (Grewe et al., 2011; Huron and Margulis, 2010). The praise for revolutionary history and heroism within red music aesthetically engages listeners while reinforcing reverence for collective ideals. Importantly, the awe elicited by red music often extends beyond individual experience into a shared collective phenomenon. Awe of this kind is a prototypical collective emotion that commonly arises in communal settings such as public gatherings and concerts, where it strengthens collective consciousness and increases feelings of belonging and pride (Shiota et al., 2003, 2017). Such collective experiences may contribute to higher subjective wellbeing.

Building on the collective and historical characteristics of red music, which emphasize shared struggle and communal identity, not all positive emotions elicited by music are equally consistent with the collective and prosocial features of red musical identity. Awe is suited to this context because it is other-oriented and self-transcendent, reducing self-focus and fostering attention to collective welfare, thereby increasing generosity and prosocial behavior (Shiota et al., 2007; Prade and Saroglou, 2016; Stellar et al., 2017; Li Y. et al., 2024). Other positive emotions such as pride and nostalgia may also be elicited by music. While other positive emotions such as pride and nostalgia may also be elicited by music, their effects are often more self-referential or retrospective, focusing on personal achievement or past experiences (Tracy and Robins, 2007; Sedikides and Wildschut, 2016).

This make them less directly connected to the immediate prosocial engagement elicited by red music. Taken together, these distinctions suggest that awe may be particularly relevant among the positive emotions elicited by red music for supporting collective-oriented and prosocial engagement. This pattern aligns with prior research emphasizing awe’s capacity to translate emotional experiences into prosocial behaviors while reflecting a collective-oriented nature within cultural contexts (Piff et al., 2015; Stellar et al., 2017; Bai et al., 2017). Therefore, it is reasonable to expect that red musical identity can foster the experience of awe. Accordingly, this study proposes Hypothesis 2: Awe at Time 2 mediates the relationship between red musical identity at Time 1 and subjective wellbeing at Time 3.

### 1.4 The mediating role of prosocial behavior

Throughout human evolution, music has played a significant role in aiding social bonding, which provides insights into its evolutionary significance (Li J. P. et al., 2024; Savage et al., 2021). Some individuals engage in altruistic or socially beneficial behavior such as helping, sharing, donating, and volunteering. In psychology, such behavior is referred to as “prosocial.” This term specifically denotes positive actions undertaken by individuals that benefit others and society at large. Examples include helping, sharing, donating, cooperating, and participating in volunteer services (Gao et al., 2021). One study has suggested that individuals are more likely to engage in helping behavior in groups with a strong social identity (Grimalda et al., 2023). Previous research has also demonstrated that individuals are more likely to display prosocial behavior toward teammates and opponents when a group’s social identity is stronger (Bruner et al., 2014). Furthermore, individuals tend to exhibit prosocial behavior more frequently toward members of their own group than toward members of other groups (Hackel et al., 2017).

When the public listens to red music, it may enhance prosocial behavior. Tourists often experience feelings of awe during red tourism. Cognitive and behavioral changes triggered by this emotion can motivate individuals to transcend their self-interest, foster prosocial behavior, and facilitate deeper information processing (Liu, 2023). Additionally, North et al. (2004) explored how different styles of music impact helping behavior. They found that participants who listened to positive and uplifting music were more willing to hand out flyers at the gymnasium than those who listened to irritating music. Further research has shown that music with prosocial lyrics can promote prosocial cognition and empathy, and encourage helpful behavior (Greitemeyer, 2013). These findings emphasize the importance of music as a means of fostering social bonds throughout human evolution. In a contemporary context, red songs hold significant political, moral, and economic value. They help individuals establish noble ideals and goals, further national self-esteem (Wang, 2017), and provide direct motivation for promoting prosocial behavior and subjective wellbeing. A red musical identity may boost subjective wellbeing by fostering prosocial connections among individuals. Based on this, this study proposes Hypothesis 3: Prosocial behavior at Time 2 mediates the relationship between red musical identity at Time 1 and subjective wellbeing at Time 3.

### 1.5 The chain mediating effect of awe and prosocial behavior

Awe is a positive social emotion that represents a profound emotional experience with the capacity to shape an individual’s inner world and perceptions of the environment. It has been demonstrated to broadly facilitate prosocial tendencies. Regardless of whether prosocial behaviors primarily involve helping or sharing, numerous experimental studies support the effect of awe upon helping behavior (Guan et al., 2019; Piff et al., 2015; Rudd et al., 2012). As a reflection of social and historical culture, music has played a significant role in both the survival and development of human societies and individual psychological development (Chen, 2020). Savage et al. (2021) proposed the “music-social bonding” theory, which suggests that music can enhance interpersonal closeness and strengthen group cohesion by coordinating individual emotions, feelings, behavior, or perspectives. Research has indicated that positive appraisals of prosocial music are correlated with an increase in prosocial behavior (Ruth, 2017). The emotional content of red music is characterized by its strong social nature and sense of group belonging. The deep social connotations and attributes of red music have been identified as key elements in identity formation, with the resultant spiritual motivation and sense of achievement being significant factors in self-realization (Zheng, 2021). One important way for college students in the new era to build cultural confidence is to promote red music culture. This not only helps reinforce their aesthetic appreciation skills, but also strengthens their sense of belonging, identity, dignity, and pride in Chinese culture, which is highly relevant in today’s context (Shen, 2020).

From a historical perspective, red music, which embodies both the intrinsic qualities of music and its cultural attributes, holds both cultural and spiritual significance. Red music songs convey artistic expression, ideological depth, popular appeal, and a revolutionary spirit. These play a vital role in helping the public cultivate a correct understanding of history, national identity, and core values, thereby developing their sense of identity with their nation. This strengthened sense of identity in turn contributes to greater subjective wellbeing. Anderson et al. (2018) induced awe by creating a simulated rafting scenario in which participants completed a 14 days diary. They found that both extraordinary and everyday experiences of nature elicited awe more effectively than other positive emotions, and that this sense of awe significantly enhanced participants’ subjective wellbeing. Similarly, Gordon and Chen (2016) provided physiological evidence that awe has positive emotional effects: experiencing awe reduces irritability and activates the parasympathetic nervous system. These findings suggest that awe can generate positive effects at both psychological and physiological levels. It helps individuals focus more deeply on their emotional experiences, increases enthusiasm for and engagement with life, and heightens subjective wellbeing. Listening to red music may similarly inspire feelings of awe, which can further promote prosocial behavior and contribute to improved subjective wellbeing. Accordingly, the present study proposes the hypothesis that awe and prosocial behavior at Time 2 sequentially mediates the relationship between red musical identity at Time 1 and subjective wellbeing at Time 3.

## 2 Materials and methods

### 2.1 Participants

A convenience sampling method was adopted to distribute and collect 750 questionnaires at baseline assessment (Time 1; T1) from college students in a particular province and city, using the Wenjuanxing platform. After excluding questionnaires with missing values, 700 valid responses were retained with an effective response rate of 93.3%. Among these, 600 participants completed the second data collection (Time 2; T2) after 6 months, and 579 participants completed the third data collection (Time 3; T3) after another 6 months. The attrition rates were 14.29% at T2 and 3.5% for T3. The final sample comprised 447 female (77.2%) and 132 male students (22.8%). The gender imbalance may be attributed to the sample being drawn from a liberal arts university, where female students typically outnumber male students. The participants were aged 18–24 years, with an average age of 20.16 years (SD = 1.09). Other demographic information can be found in Supplementary Table 1.

To examine whether sample attrition introduced systematic bias, we conducted attrition analyses by comparing participants who completed all three waves of data collection (*n* = 579) with those who dropped out at either T2 or T3 (*n* = 121). Independent-samples *t*-tests were performed on key baseline variables including red musical identity, awe, prosocial behavior, and subjective wellbeing. The results showed no significant differences between the two groups (all *ps* > 0.05), suggesting that attrition did not systematically bias the sample. Therefore, the final sample retained for analysis may be considered representative of the initial baseline sample.

The measurements were conducted by a trained research assistant with students in a regular classroom setting during school hours. There was no time limit; generally, participants took approximately 20 min to complete the questionnaire. All participants signed an informed consent form before completing the survey (and during the online survey). Participants could withdraw from the study at any time, and were guaranteed anonymity and confidentiality; only the researchers had access to the questionnaire. However, as the sample consisted solely of university students from a single province and city, the generalizability of the findings may be limited. This study was approved by the Ethics Committee of the Department of Psychology at Southwest University.

### 2.2 Research design

The present study employed a three-wave longitudinal panel design with a 6 months interval between each wave. Collins (2006) recommends that when the number of waves is relatively small (≤ 8), intervals of at least 6 months help capture meaningful psychosocial changes and reduce short-term fluctuations. This choice is also consistent with prior longitudinal studies regarding prosocial behavior (Liu et al., 2020; Xu et al., 2025), awe (Ma et al., 2024; Skalski-Bednarz et al., 2024), and subjective wellbeing (Zhou et al., 2020; Luo, 2024). Although direct longitudinal evidence for red musical identity is lacking, related constructs such as musical and cultural identity have demonstrated both stability and stage-specific variability (Evans and McPherson, 2014). Consequently, a 6 months interval was adopted in the present study, based on these methodological considerations and empirical findings.

### 2.3 Measures

#### 2.3.1 Red musical identity

Our research team developed the Red Musical Identity Scale based on an extensive literature review, preliminary qualitative research, and established theoretical frameworks. Item generation was informed by Social Identity Theory, which conceptualizes identity in cognitive, affective, and behavioral terms (Tajfel and Turner, 1986), and by Phinney’s (1990) model of ethnic identity development, which views identity as a multidimensional construct encompassing belonging, positive evaluation, cultural interest, and active participation. In the Chinese context, cultural identity, which is rooted in shared historical narratives and values, has been demonstrated to influence attitudes, emotions, and behavior related to social engagement (Du et al., 2025). In addition, empirical sources were consulted in item design, including the Musical Identity Measure (Burland et al., 2022), the Multigroup Ethnic Identity Measure (Phinney, 1992), and the Red Cultural Identity Questionnaire for Contemporary College Students (Fang et al., 2019).

It was designed to measure the degree of identification with red music and political or historical themes. The scale contains 15 items, including three subdimensions: cognitive-emotional, cultural and behavioral identity. Item development was conducted through expert interviews, focus group discussions, and pre-testing to ensure good psychometric properties. Expert consultation involved five specialists, three in music education and red cultural studies, and two in psychology and measurement, who were selected according to their academic expertise, publication record, and familiarity with identity research in Chinese cultural contexts. Focus group discussions with target users were conducted to ensure conceptual clarity and cultural relevance.

Pilot testing was conducted with an independent sample of 40 college students, distinct from the main study sample, to assess item clarity, response distribution, and preliminary reliability. Feedback on participants’ understanding of each item was collected, and items showing ambiguity or comprehension difficulties were further discussed and revised with the expert team, resulting in the final version of the Red Musical Identity Scale for formal use. The scale is suitable for people aged 18 years and above, and is scored using a five-point Likert scale. Higher scores indicate stronger identification with red music. The scale showed good internal consistency in a preliminary study (Cronbach’s α = 0.95). To evaluate the psychometric properties of the Red Musical Identity Scale, we recruited an independent cross-sectional validation sample (*N* = 3,456) was recruited that did not overlap with the longitudinal panel used for the main analyses. As evidence of criterion-related validity, scores on the Red Musical Identity Scale showed positive correlations with awe (Dispositional Positive Emotion Scale; DPES), prosocial behavior (Prosocial Tendencies Measure; PTM), subjective wellbeing (Subjective Happiness Scale; SHS), and positive affect (Positive and Negative Affect Schedule – Positive Affect subscale; PANAS-PA), all *p*s < 0.05 (*N* = 3,456; see Supplementary Table 2). To examine the psychometric structure of the Red Musical Identity Scale, we randomly split the total sample (*N* = 3,456) into two subsamples. Subsample 1 (*n* = 1,724) was used for exploratory factor analysis (EFA), which revealed a three-factor solution. Subsample 2 (*n* = 1,732) was used to conduct confirmatory factor analysis (CFA). The CFA results indicated a good model fit: χ^2^ = 1153.181, df = 84, χ^2^/df = 13.73, CFI = 0.973, TLI = 0.966, SRMR = 0.024, RMSEA = 0.086. It is important to note that the EFA supported a three-factor structure, which we have adopted as the final version of the Red Musical Identity Scale (Cronbach’s α = 0.97 at T1, 0.98 at T2, and 0.97 at T3). Although the χ^2^/df ratio was high, this statistic is highly sensitive to large sample sizes and often inflates misfit in very large datasets (Hu and Bentler, 1995; Hair et al., 2014). Similarly, the RMSEA value was at the upper boundary of acceptability (Shi et al., 2022), but given the excellent CFI, TLI, and SRMR values, the overall model fit can still be considered adequate.

#### 2.3.2 Awe

Awe was assessed using the awe subscale (DPES-awe) of the DPES compiled by Shiota et al. (2006) and translated and revised by Dong et al. (2013). The DPES includes seven subscales: joy, pride, compassion, contentment, love, amusement, and awe. The DPES-awe includes six items and uses a seven-point rating, ranging from 1 (strongly disagree) to 7 (strongly agree). The higher the score, the higher the individual’s tendency to feel awe. The internal consistency coefficient of the scale in this study was 0.92 at T1, T2, and T3.

#### 2.3.3 Prosocial behavior

The PTM compiled by Carlo and Randall (2002) and translated and revised by Kou et al. (2007) was used. This scale measures individuals’ prosocial tendencies. It contains 26 items across six dimensions: anonymous, public, compliant, altruistic, dire, and emotional. Different dimensions reflect the possibility of an individual performing prosocial behavior in a situation. The scale uses a five-point rating (1 = describes me greatly; 5 = does not describe me at all). The higher the scale score, the higher the individual’s tendency to engage in prosocial behavior. The internal consistency coefficient of the scale in this study was 0.98 at T1, T2, and T3.

#### 2.3.4 Subjective wellbeing

The SHS compiled by Lyubomirsky and Lepper (1999) was used for measurement. This scale contains four items that examine an individual’s judgment of his or her subjective wellbeing. The scale uses a seven-point scoring system (1 = very unhappy; 7 = very happy). The higher the score, the higher the individual’s subjective wellbeing level. Question 4 was reverse scored. The scale’s internal consistency coefficients in this study were s 0.73, T1, 0.78 at T2, and 0.75 at T3.

### 2.4 Statistical analysis

All data were analyzed using SPSS 25.0 to examine common method bias, descriptive statistics, correlation analysis, and reliability analysis. To investigate the sequential mediating effects of awe and prosocial behavior at T2 on the relationship between red musical identity at T1 and subjective wellbeing at T3, a mediation analysis was conducted using Model 6 of the PROCESS macro (version 3.4) for SPSS, following the procedure outlined by Fang et al. (2012). All data were standardized first. The significance of the regression coefficient was analyzed using the bootstrap method (repeated sampling 5,000 times).

## 3 Results

### 3.1 No serious common method bias

First, this study included all items in each questionnaire in SPSS 25.0 and used exploratory factor analysis to test for possible common method bias (Zhou and Long, 2004). The results showed that there were 19 factors with characteristic roots greater than 1, and the first common factor explained 32.86% of the variance, which was less than 40%. Therefore, there was no serious common method bias.

### 3.2 Gender differences and significant correlations

First, an independent-samples *t*-test was conducted to test for sex differences. Male students scored significantly lower than female students on red musical identity at T1 (*t* = −2.18, *p* = 0.03). However, there were no significant sex differences in awe at T2 (*t* = 0.60, *p* = 0.552), prosocial behavior at T2 (*t* = 0.73, *p* = 0.464), or subjective wellbeing at T3 (*t* = 0.00, *p* = 0.998).

Descriptive information for the main variables and their correlation coefficients is presented in Table 1. The results showed that red musical identity at T1 was significantly positively correlated with subjective wellbeing at T3 (*r* = 0.28, *p* < 0.01). Furthermore, red musical identity at T1 was significantly positively correlated with awe at T2 (*r* = 0.28, *p* < 0.01) and prosocial behavior at T2 (*r* = 0.29, *p* < 0.01). Awe at T2 was significantly positively correlated with prosocial behavior at T2 (*r* = 0.68, *p* < 0.01) and subjective wellbeing at T3 (*r* = 0.34, *p* < 0.01). These results provide a basis for testing the multiple mediation effects.

**TABLE 1:** Descriptive statistics and Pearson correlations for all measures.

Variables	M	SD	1	2	3	4
1. Red musical identity at T1	93.50	12.38	1.00	–	–	–
2. Awe at T2	33.71	5.91	0.28**	1.00	–	–
3. Prosocial behavior at T2	104.25	15.47	0.29**	0.68**	1.00	–
4. Subjective wellbeing at T3	20.83	3.65	0.28**	0.34**	0.36**	1.00

*N* = 579; **p < 0.01.

### 3.3 Red musical identity at t1 predicts subjective wellbeing at T3 via awe and prosocial behavior at T2

Table 2 presents the regression analysis results. After controlling for sex, awe, and prosocial behavior at baseline, red musical identity at T1 significantly positively predicted subjective wellbeing at T3 (β = 0.19, *p* < 0.001). After adding the two mediating variables, red musical identity at T1 significantly positively predicted awe at T2 (β = 0.12, *p* < 0.05); awe at T2 significantly positively predicted prosocial behavior at T2 (β = 0.60, *p* < 0.001) and subjective wellbeing at T3 (β = 0.17, *p* < 0.01); prosocial behavior at T2 positively predicted subjective wellbeing at T3 (β = 0.16, *p* < 0.01); and red musical identity at T1 still significantly predicted subjective wellbeing at T3 (β = 0.15, *p* < 0.01).

**TABLE 2:** Results of the regression analysis of study variables.

Path	Regression equation		Overall fitting coefficient			Regression coefficient and significance		
	DV	IV	*R*	*R* ^2^	*F*	*B*	95% CI	*t*
a	Awe (T2)	Red musical identity (T1)	0.41	0.17	21.7222	**0.12**	(0.0182, 0.2206)	2.3175*
b1	Prosocial behavior (T2)	Red musical identity (T1)	0.72	0.51	95.3180	0.04	(−0.0423, 0.1135)	0.8980
		Awe (T2)	–	–	–	**0.60**	(0.5342, 0.6629)	18.2725***
b2	Subjective wellbeing (T3)	Red musical identity (T1)	0.43	0.19	17.8389	**0.15**	(0.0518, 0.6629)	2.9555**
		Awe (T2)	–	–	–	**0.17**	(0.0639, 0.2789)	3.1311**
		Prosocial behavior (T2)	–	–	–	**0.16**	(0.0526, 0.2739)	2.8979**

After adjusting for age, sex, awe, and prosocial behavior at baseline. Path a represents the effect of red musical identity (T1) on awe (T2). Path b1 represents the effects of red musical identity (T1) and awe (T2) on prosocial behavior (T2). Path b2 represents the effects of red musical identity (T1), awe (T2), and prosocial behavior (T2) on subjective wellbeing (T3). 95% CI refers to the 95% bias-corrected bootstrap confidence interval (based on 5,000 resamples). All regression results are adjusted for age, sex, and baseline levels of awe and prosocial behavior. ***p < 0.001; **p < 0.01; *p < 0.05. DV, dependent variable; IV, independent variable. Bold values indicate that the corresponding path is significant.

**FIGURE 1:**
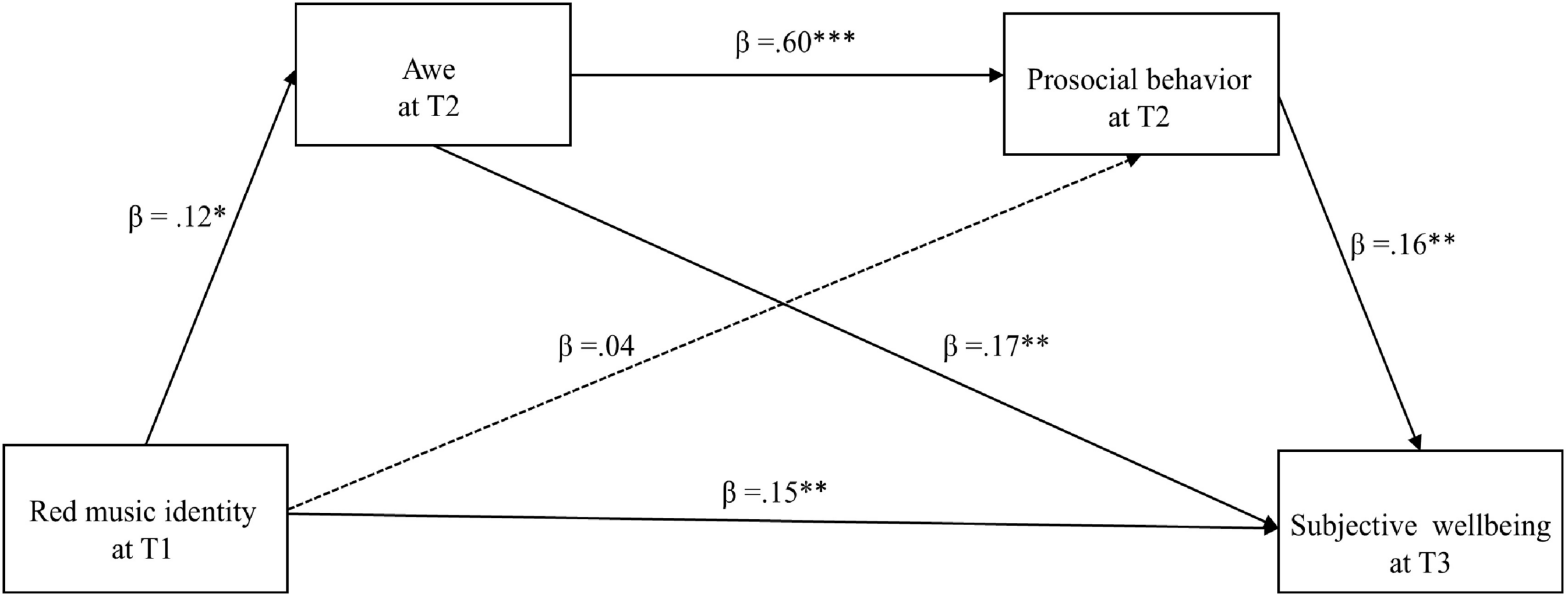
A chain mediation model of awe at T2 and prosocial behavior at T2 between red musical identify at T1 and subjective wellbeing at T3. Beta values represent the standardized path coefficients. ***p < 0.001; **p < 0.01; *p < 0.05. Dashed lines represent non-significant paths (e.g., Red musical identity→prosocial behavior).

Table 3 presents the mediating effects based on the bootstrapping method. The bootstrap confidence intervals of the above mediation effects did not contain a value of zero, indicating that the three mediation effects reached significance. Specifically, three paths produced mediating effects: the indirect effect 1 (0.02) of red musical identity at T1→awe at T2→subjective wellbeing at T3 path; the indirect effect 2 (0.01) of red musical identity at T1→prosocial behavior at T2→subjective wellbeing at T3 path; and the indirect effect 3 (0.01) of red musical identity at T1→awe at T2→prosocial behavior at T2→subjective wellbeing at T3 path. Table 3 shows that the three indirect effects accounted for 9.98%, 3.54%, and 6.36% of the total effects, respectively. Figure 1 presents the two-mediator sequential model. To provide a concise overview of all hypothesis testing results, Table 4 summarizes whether each proposed hypothesis was supported.

**TABLE 3:** Results of sequential mediation analysis.

Indirect effect path	Effect	Boot SE	Boot 95% CI	Ratio of indirect to total effect of X on Y
Indirect effects of X on Y	0.04	0.0176	(0.0052, 0.0743)	78.01%
Ind 1 (X→M1→Y)	0.02	0.0122	(0.0011, 0.0490)	9.98%
Ind 2 (X→M2→Y)	0.01	0.0066	(−0.0061, 0.0203)	3.54%
Ind 3 (X→M1→M2→Y)	0.01	0.0072	(0.0004, 0.0282)	6.36%

After adjusting for age, sex, awe, and prosocial behavior at baseline. X, red musical identity at T1; M1, awe at T2; M2, prosocial behavior at T2; Y, subjective wellbeing at T3.

**TABLE 4:** Summary of hypothesis testing results.

Hypothesis	Relationship	Result
H1	Red musical identity at Time 1 positively and significantly predicts subjective wellbeing at Time 3.	Supported
H2	Awe at Time 2 mediates the relationship between red musical identity at Time 1 and subjective wellbeing at Time 3.	Supported
H3	Prosocial behavior at Time 2 mediates the relationship between red musical identity at Time 1 and subjective wellbeing at Time 3.	Not supported
H4	Awe and prosocial behavior at Time 2 sequentially mediate the relationship between red musical identity at Time 1 and subjective wellbeing at Time 3.	Supported

## 4 Discussion

This study examined the relationship between red musical identity and subjective wellbeing using a three-wave longitudinal design to examine the mediating roles of awe and prosocial behavior. The sample was drawn from a single university in western China and was predominantly female (77.2%), which means that these findings are most directly applicable to similar student populations. The results revealed that red musical identity measured at T1 predicted subjective wellbeing at T3 through a sequential indirect pathway involving awe (at T2) and prosocial behavior (at T2). Music is a powerful tool for emotional expression, and it can help convey emotional communication (Yang and Zheng, 2021). If red music is found to contribute to individuals’ subjective wellbeing, this would further highlight its potential artistic and educational value.

### 4.1 Effect of red musical identity at T1 on subjective wellbeing at T3

University students’ red musical identity at T1 was positively associated with their subjective wellbeing at T3, thus support Hypothesis 1. This result is similar to that of a previous study (Park et al., 2025). As a key dimension of red musical identity, cultural identity has been comprehensively conceptualized in the existing literature. Scholars have analyzed this from both the macro and micro perspectives. At the macro level, cultural identity comprises national and ethnic identities, while at the micro level, it is a multidimensional construct involving psychological traits such as attitudes, cognition, and emotions. Cultural identity exists not only at the societal level, but also at the individual level, serving as the basis for individuals to make cultural judgments and self-positioning decisions in various contexts (Dong et al., 2014). Music, refined through long-term integration among various ethnic groups, has become a cultural symbol associated with national character and fosters a sense of identity (Bai, 2024). Research has indicated that red songs, with their uncomplicated narratives and emotionally charged lyricism, can evoke memories of arduous yet passionate revolutionary times in those who sing them. The compelling power of red songs, as demonstrated by their melodies and lyrics, promotes ideological education and may strengthen university students’ identity with the songs (Tao, 2011). Moreover, various studies support the proposition that red tourism—in essence, a spiritual and cultural product analogous to red music—has been associated with national belonging and subjective wellbeing (Chen and Liu, 2020; Jiang et al., 2021).

Social identity theory posits that individuals derive a sense of self from the social groups to which they belong (Tajfel and Turner, 1979). In this study, red music, as an important medium for transmitting collective memory and culture values, may strengthen the listeners’ identity with their nation and ethnic group. Such collective identity has been linked to self-esteem, belonging, and security, which are in turn associated with greater subjective wellbeing (Hachem and Toro, 2022). Some studies have integrated the stimulus-organism-response (S-O-R) framework with the broaden-and-build theory of positive emotions and the prototype theory of awe to construct a conceptual model of red tourists’ subjective wellbeing. The findings suggest that perceptions in red tourism can evoke red memories, which in turn either directly or indirectly improve tourists’ subjective wellbeing (Yan et al., 2023). Therefore, we propose that individuals who engage with red music, through studying, listening, or performing, tend to experience greater subjective wellbeing when they have a stronger sense of identity and belonging with red music culture. This sense reflects individuals’ recognition of the ideological and cultural achievements that emerged throughout the nation’s development. This includes both the acceptance and promotion of red cultural resources as part of a shared historical memory, and the dissemination of red culture, which is significant in the contemporary era (Hao, 2022). To further explore the wellbeing implications of red musical identity, red music could be integrated into educational and cultural initiatives, such as curricula, festivals or community events. Such practices may provide opportunities for aesthetic engagement and social connectedness, which could in turn be linked to higher levels of self-esteem, belonging, and wellbeing.

### 4.2 Awe at T2 as a mediator between red musical identity at T1 and subjective wellbeing at T3

The results of this study indicated that awe at T2 mediated the relationship between red musical identity and subjective wellbeing at T3, thereby support Hypothesis 2. Artistic expressions, music, and natural wonders are known to elicit a range of emotional responses, among which awe is particularly common (Keltner and Haidt, 2003). Previous research comparing the frequency and intensity of awe experienced by art and non-art students found that art students reported significantly higher levels of awe in terms of both frequency and intensity. Furthermore, awe was positively associated with subjective wellbeing, a finding that aligns with previous research (Dong, 2016). According to the broaden-and-build theory of positive emotions, awe has been to produce beneficial effects both psychologically and physiologically. It may encourage individuals to develop greater passion for and engagement in life, may facilitate cognitive transformation, may strengthen immune system function, may improve the quality of social relationships, and may ultimately contribute to subjective wellbeing (Guo and Wang, 2007). In the context of red music, the emotion of awe may encourage individuals to experience a broader sense of connection with history, culture, and even larger social collectives. This deepened sense of connection may create spiritual fulfilment and may foster greater social and cultural engagement, both of which are important sources of subjective wellbeing. For instance, red music education can be promoted using digital technologies such as virtual reality (VR) and augmented reality (AR), which may enhance may enhance the vividness and emotional resonance of red music education. These technologies allow students to revisit historical scenes in simulated environments, thereby strengthening the vividness and emotional resonance of red music education. Through this multisensory engagement, learners are not only able to appreciate the artistic value of red music, but may also experience a sense of awe relating to its cultural and historical contexts. This emotional immersion may help cultivate a deeper sense of national identity and may contribute to students’ emotional development.

### 4.3 Prosocial behavior at T2 as a non-significant mediator between red musical identity at T1 and subjective wellbeing at T3

The analyses results did not support Hypothesis 3, indicating that prosocial behavior at T2 did not mediate the relationship between red musical identity at T1 and subjective wellbeing at T3, which is inconsistent with previous findings (Hong et al., 2023). One explanation for this phenomenon is that musical identity involves distinct psychological mechanisms. Specifically, red musical identity reflects an individual’s acceptance of and emotional attachment to red music at the cultural, cognitive, emotional, and behavioral levels. The focus is on fostering an understanding of the historical values, national spirit, and patriotic sentiments embodied in music. Nevertheless, such identification is likely to function mainly as a personal attitude that does not consistently translate into observable prosocial behavior. The expression of behavior necessitates not only identity, but also the application of rational decision-making, which are further influenced by external situational triggers, intrinsic motivation, social norms, and societal values (Cardona-Isaza et al., 2023; Chui et al., 2025). For instance, previous studies have suggested that participation in group music activities may foster prosocial behavior by enhancing social bonding and peer support, while social norms may further encourage individuals to engage in prosocial actions and maintain positive social relationships (Yang et al., 2025; Van den Bos et al., 2018). Therefore, in the absence of external incentives or contextual support, identification alone may not reliably predict the occurrence of prosocial behavior. In addition, the nature of red music itself needs to be considered in this context. The core of red music lies in the inheritance of history and spirit. Its primary function is to disseminate cultural values and construct collective identity, rather than to directly elicit everyday prosocial behavior. Drawing upon theories of collective and cultural memory (Halbwachs, 1992/1925; Assmann, 2011), historical experiences are preserved and transmitted through rituals, symbols, and artistic forms, with music serving not only as an aesthetic medium but also as a vehicle for the construction of social identity and belonging (Turino, 2008). Such functions are most evident in commemorative atmospheres and collective practices (Sumartojo, 2016), where ritualized spaces and shared participation reinforce historical memory and national identity. The outcomes of these practices may primarily involve the strengthening of symbolic solidarity and group cohesion, rather than the immediate and universal manifestation of prosocial behavior. Accordingly, the influence of red music on prosocial behavior is more likely to be context-dependent and temporally delayed (Anderson, 2006; Páez et al., 2015), as exemplified in collective commemorative activities. Moreover, the developmental stage of university students may have impacted the findings. Prior studies have suggested that early childhood and elementary school as critical periods when music interventions more effectively foster prosocial tendencies (Martí-Vilar et al., 2023). In contrast, university students tend to have more stable values and greater autonomy, which makes the translation from cognitive and emotional identification to observable behaviors less direct. Variations in exposure duration and frequency may also weaken this pathway. For example, short-term interventions such as the 1 year “El Sistema” program in Venezuela showed limited effects (Alemán et al., 2017), whereas evidence suggest that longer and more consistent engagement may be required to produce measurable behavioral or neural changes (Kraus et al., 2014).

Beyond these contextual and developmental explanations, a broader theoretical framework provides deeper insight into the observed non-significant pathway. In this study, Hypothesis 3 was not supported, a finding that may be interpreted as consistent with the mechanism proposed in the Music Processing Model (Ruth, 2019). This model suggests that the influence of music on individual behavior often does not occur directly but may be mediated through the processing of an internal state, in which music, personal input, and situation input interact to activate cognitive, affective, and arousal states that subsequently drive behavior. In the context of this study, red musical identity may be understood as a psychological resource shaped by both music and personal input, whose impact on prosocial behavior likely depends on the activation of internal affective states. Thus, the absence of a direct effect of red musical identity on prosocial behavior is consistent with the model’s emphasis on the necessity of internal state mediation. It is possible that only when red music evokes an emotional experience such as awe can it further promote prosocial actions. This finding may suggest that the pathway from red musical identity to behavior aligns more closely with the indirect influence mechanism described by the Music Processing Model rather than a direct effect. Future research, particularly with more diverse and representative samples, could examine which emotional states, such as awe, pride, or collective empathy, serve as key mediators in this process, as well as how different situational contexts amplify or dampen these emotional triggers, thereby deepening our understanding of the sequential pathway linking musical identity, emotional experience, and prosocial outcomes.

### 4.4 The chain mediating role of awe and prosocial behavior at T2 in the relationship between red musical identity at T1 and subjective wellbeing at T3

The findings indicated that awe and prosocial behavior at T2 serve as sequential mediators in the relationship between red musical identity at T1 and subjective wellbeing at T3. Greater experiences of awe may promote prosocial behavior, which in turn, may be associated with individuals’ subjective wellbeing. Music has been identified as a common trigger for awe (Keltner and Haidt, 2003; Konečni, 2008; Yaden et al., 2019). Red music, as a cultural expression that emerged during a specific period of modern Chinese history, has been described as serving as a foundation for national unity and collective progress and as an essential psychological pillar supporting the existence of both the state and the Chinese nation (Jiang and Zhang, 2009; Zuo and Wen, 2017). Thus, uplifting lyrics and inspiring melodies in red music may stir feelings of awe. Moreover, previous empirical research has suggested that awe is associated with greater prosocial behavior (Prade and Saroglou, 2016). For instance, awe may increase individual generosity, helping behavior, and moral action (Huang, 2018; Piff et al., 2015), and reduce aggressive behavior (Yang et al., 2016). Research has also shown a positive correlation between awe and subjective wellbeing. Awe may not only encourage prosocial behavior but also appears to play a role in explaining and predicting subjective wellbeing. The generation of awe may be important for assisting an individual’s subjective wellbeing and overall physical and mental health (Dong, 2016). Thus, red music may elevate individuals’ sense of identity and feelings of awe, which in turn may foster prosocial behavior, thereby potentially contributing to individuals’ subjective wellbeing. This process may illustrate the potential role of music in boosting social connection and mental health, and may align with the music-social bonding theory, which suggests that music can strengthen social cohesion by harmonizing people’s emotions and behavior (Savage et al., 2021). As an important medium for inheriting and promoting revolutionary culture, red music conveys people’s emotions toward their country that cannot be expressed in words. It encompasses themes of life, death, love, and peace, as well as the efforts made by revolutionary ancestors to achieve national independence and universal liberation. In addition, it may play an important role in contemporary youth with history, and plays an irreplaceable role in contemporary education. Although the chained mediating effect of awe via prosocial behavior on subjective wellbeing accounted for only 6.36% of the total effect, this modest proportion may represent a meaningful pathway. It suggests that, within the present predominantly female, single-site student sample, red music may enhance happiness more effectively when emotional arousal is coupled with behavioral practices that channel awe into concrete prosocial actions. Prior research has indicated that joint musical activities such as choir singing, ensemble performances, and collaborative music-making may foster cooperation and helping through interpersonal synchronization and positive social interaction (Kirschner and Tomasello, 2010; Williams et al., 2015; Li J. P. et al., 2024). Incorporating participatory red music activities (e.g., red-themed choirs or concerts) into educational and community contexts may therefore strengthen the translation of awe into prosocial behavior and potentially contribute to improvements in wellbeing. Practical implications can also be drawn from these findings. Beyond provoking awe and prosocial behavior, red music may be further integrated into aesthetic education through innovative practices that combine artistic creation with collaborative participation, such as red-themed musical psychodramas, short films, or virtual reality assisted historical re-enactments. These practices extend the potential influence of red music from emotional stimulation to long term educational outcomes, offering students opportunities for critical reflection, creative expression, and collective engagement. As such, red music may not only contributes to prosocial behavior and subjective wellbeing, but also may strengthen its broader educational and cultural value.

### 4.5 Limitations and future research

This study developed a chain mediation model to explore the potential mechanism through which red musical identity may be associated with college students’ subjective wellbeing. The results may provide valuable guidance for aesthetic education and mental health initiatives in higher education institutions. However, the study does hold certain limitations. First, it relied primarily on self-report measures, which may be subject to social desirability bias. Future research could employ experimental methods or other objective assessments of musical behavior. Second, the sample size was relatively small; larger samples should be used in future studies to obtain more robust evidence. Third, the sample consisted solely of university students from a city in western China, which may limit the generalizability of the findings. In addition, the sample was predominantly female (77.2%) due to recruitment from a liberal arts university, which may further reduce representativeness. Future research should recruit more diverse and gender-balanced samples to increase generalizability. Fourth, potential confounding variables were not fully controlled. In particular, students’ participation in political or cultural associations, prior exposure to red culture education, and family cultural capital may have influenced both their musical identity and wellbeing. These background factors could partially account for the observed relationships, because they shape students’ opportunities for musical engagement and broader cultural values. Future research should control for such variables through targeted survey items or stratified sampling in order to obtain more precise estimates of the unique effects of red music. Finally, although the longitudinal survey design captured naturally occurring variations over time, it cannot provide the same level of causal inference as experimental methods. Future studies could therefore adopt experimental or mixed-method designs to more rigorously test the potential causal mechanisms proposed in the study.

This study may offer a scientific basis for the formulation and implementation of cultural policies. It incorporates awe and prosocial behavior as chain mediators, potentially providing new theoretical guidance for aesthetic education and mental health work in higher education. It also highlights the vital role of cultural education in fostering spiritual and emotional development. Red music is a significant component of Chinese culture and may play a vital role in cultivating young people’s national identity and social responsibility. Within the Chinese cultural context, university students develop a stronger sense of national and ethnic identity through red music, which may in turn foster a heightened sense of awe. These positive emotional experiences may enhance students’ psychological resilience, alleviate negative emotions, and may contribute to greater subjective wellbeing. This study may not only enrich the theoretical framework of music psychology and wellbeing research, but also may offer a practical path for leveraging indigenous cultural resources to promote mental health and social harmony. These findings underscore the potential importance of further exploring the educational potential of Chinese culture. From psychological and aesthetic education perspectives, this study may provide new insights for improving collaborative educational mechanisms in universities and may ultimately contributing to the goal of holistic student development and positioning culture as a supportive force in strengthening social cohesion and public wellbeing.

## 5 Conclusion

This study suggests a potential role of red musical identity in relation to college students’ subjective wellbeing. The findings indicate that red musical identity measured at T1 significantly and positively predicted subjective wellbeing at T3. Furthermore, awe experienced at T2 served as a significant independent mediator from red musical identity at T1 to subjective wellbeing at T3. Additionally, the sequential mediating effect of awe and prosocial behavior at T2 was significant. Red musical identity at T1 showed indirect effects on subjective wellbeing at Time 3 successively via the awe at Time 2 and prosocial behavior at Time 2. These results may underscore the psychological significance of culturally rooted music and may provide practical implications for incorporating red music into aesthetic education and mental health promotion strategies in higher education settings. Given that the sample was drawn from a single university in western China and was predominantly female (77.2%), these findings should be interpreted with caution, and further studies with more diverse and representative samples are warranted to confirm their generalizability.

## Data Availability

The original contributions presented in this study are included in this article/Supplementary material, further inquiries can be directed to the corresponding author.
