# Supplementary material for: Red musical identity and subjective wellbeing: a longitudinal study of the chain mediating roles of awe and prosocial behavior
Source: Front Psychol. 2025 Oct 6;16:1635179. doi: 10.3389/fpsyg.2025.1635179 (PMC12536728; doi:10.3389/fpsyg.2025.1635179)
Supplement: Supplementary file 1 [file Table_1.docx]

**Table S1.** Population characteristics of the study. (N = 579).

| Characteristics | N | % |
| --- | --- | --- |
| **Age (years) (Mean±SD)** | 20.16±1.09 | / |
| **Gender** |  |  |
| Males | 132 | 22.8 |
| Females | 447 | 77.2 |
| **Mother’s education level** |  |  |
| Until middle school | 328 | 56.6 |
| Technical secondary school or high school | 179 | 30.9 |
| College | 1 | 0.2 |
| Master or above | 71 | 12.3 |
| **Father’s education level** |  |  |
| Until middle school | 223 | 38.5 |
| Technical secondary school or high school | 260 | 44.9 |
| College | 2 | 0.3 |
| Master or above | 94 | 16.2 |
| **Monthly family income** |  |  |
| ＜ 2000 CNY | 72 | 12.4 |
| 2000 CNY-6000 CNY | 326 | 56.2 |
| 6000 CNY -10000 CNY | 125 | 21.6 |
| ＞ 10000 CNY | 56 | 9.7 |

Note. Values are presented as n (%) unless otherwise indicated. Age is presented as Mean ± SD. Percentages may not total 100 due to rounding.

**Table S2.** The criterion-related validity of Red Music Identity Scale.

| Variables | *M* | *SD* | 1 | 2 | 3 | 4 |
| --- | --- | --- | --- | --- | --- | --- |
| 1. Red musical identity | 92.82 | 13.56 | 1.00 |  |  |  |
| 2. Awe | 33.07 | 6.91 | 0.54** | 1.00 |  |  |
| 3. Prosocial behaviour | 104.70 | 17.80 | 0.49** | 0.62** | 1.00 |  |
| 4. Subjective well-being | 20.48 | 3.86 | 0.34** | 0.46** | 0.47** | 1.00 |
| 5. Positive affects | 36.15 | 7.47 | 0.39** | 0.59** | 0.62** | 0.57** |

Note: N=3456; ***p*＜0.01.
